# Transcriptomic differences in immune- and stress-related pathways associated with artificial rearing in the endangered hog deer (*Axis porcinus*)

**DOI:** 10.1186/s12864-025-12484-y

**Published:** 2026-01-07

**Authors:** Ya Ma, Kangning Huang, Guangyao Geng, Fei Cao, Bingfu Sha, Enle Pei

**Affiliations:** Shanghai Zoo, Shanghai, 200335 China

**Keywords:** *Axis porcinus*, Artificial rearing, Transcriptome, Immune profile, Stress response

## Abstract

**Background:**

The hog deer (*Axis porcinus*), an endangered cervid species, has experienced severe population declines, making captive breeding essential for conservation. Artificial rearing has been implemented at the Shanghai Zoo, China, to improve fawn survival; however, its physiological impacts remain unclear. To elucidate the molecular consequences of different rearing modes, we conducted a comparative blood transcriptome analysis between artificially and naturally reared hog deer.

**Results:**

Whole-blood RNA sequencing of 10 hog deer individuals (six naturally and four artificially reared at the Shanghai Zoo) generated 84.3 Gb of high-quality data. De novo assembly produced 178,336 unigenes, with 40.2% annotated against the NCBI non-redundant database, mainly matching cervid species. Expression profiling and principal component analysis revealed clear segregation between groups. Differential expression analysis identified 3,045 genes (560 upregulated, 2,485 downregulated) in the artificial group. Functional enrichment showed upregulation of adaptive immune pathways, including antigen presentation (major histocompatibility complex class II molecules and CD74) and B cell activation (CD79/19 and transcription factor genes *EBF1*/*SPIB*), whereas innate immune and inflammatory responses—such as cytokine production (interleukin-6) and neutrophil activation (toll-like receptor 4/CD14)—were downregulated. Stress response modules, involving xeroderma pigmentosum complementation group C in the nucleotide excision repair pathway, also showed reduced expression, suggesting improved adaptation to captive conditions. In addition, pathways related to glutamine family amino acid and lipid metabolism were more active in the artificial group, potentially reflecting differences in early nutritional regimes.

**Conclusions:**

Our findings reveal that artificial rearing in hog deer induces a distinct transcriptomic signature, marked by a shift from innate to adaptive immunity, reduced stress responses, and altered metabolic activity. These molecular differences may underlie improved tolerance to captivity but could also compromise early pathogen detection. This study provides novel insight into the physiological consequences of artificial rearing and offers a molecular basis to refine management practices for endangered cervid conservation and reintroduction programs.

## Background

The hog deer (*Axis porcinus*)**,** a member of the Cervidae family, primarily inhabits moist tall grasslands in regions including southwestern China, India, Myanmar, and Laos [1–3]. In recent years, wild populations have declined significantly, leading to its classification as an Endangered species by the IUCN Red List in 2014 [4]. In China, the hog deer is listed in CITES Appendix I and is currently found only in the southwestern part of Yunnan Province, where it was once thought to be extinct [4–6]. Captive populations exist solely at two zoos in China, totalling about 140 individuals, with approximately 40 housed at the Shanghai Zoo. Simple sequence repeat genotyping by Geng et al. revealed a relatively high genetic diversity in the Shanghai population, with an average observed heterozygosity of 0.680 [7].

The reproductive behavior of hog deer is not fully understood, and breeding can occur year-round. Observations suggest that fawns born in winter have lower survival rates under natural conditions, worsened by inexperienced maternal care or stress-induced abandonment [8, 9]. The Shanghai Zoo has thus explored artificial rearing as a conservation strategy to enhance hog deer population growth and facilitate their reintroduction. However, artificial-reared individuals differ significantly in husbandry practices from naturally reared counterparts prior to their return to the herd. While no notable differences in behavior, morphology, or nutritional status have been observed, potential molecular-level differences remain unclear.

RNA sequencing enables comprehensive assessment of gene expression**,** offering both quantitative and global insights [10–12], and has been used widely for animal adaptation research. Surlis et al. identified immune-related pathway differences between artificially and naturally suckled calves via blood transcriptomics [13]; Similarly, Yang et al. showed that wild training in giant pandas led to upregulation of innate and cellular immunity pathways [14], and Watson et al. reported pathway enrichment differences in immunity, stress, and metabolism between great tits from urban and rural environments [15]. It is thus expected that transcriptome profiling can reveal phenotypic plasticity and environment-related changes in the absence of genetic variation, while being less susceptible to acute external influences compared to proteomics or metabolomics [16].

Despite lacking an annotated hog deer reference genome, de novo transcriptome assembly is feasible using the TRINITY software [17], and gene annotation can be performed referring to databases such as the NCBI non-redundant (NR) database. Transcriptomic analysis has been validated in hog deer: Sui et al. identified immune pathway expression responses in hog deer peripheral blood mononuclear cells after polyinosinic:polycytidylic acid stimulation [18], and Liu et al. revealed sex-specific gene expression differences via blood transcriptome sequencing [5].

In this study, we performed transcriptomic analysis on the whole blood samples from 10 hog deer at the Shanghai Zoo to evaluate the influence of different rearing methods on gene expression and individual fitness. The results aim to provide molecular insights to optimize artificial rearing strategies and inform assessments of reintroduction potential in captive populations.

## Methods

### Sample collection and RNA extraction

In March 2025, whole blood samples were collected on the same day from 10 hog deer (*Axis porcinus*) individuals at the Shanghai Zoo. All the individuals were captive animals owned and managed by the Shanghai Zoo, of which six (3 males, 3 females) were naturally reared, and four (2 males, 2 females; aged 4–14 months) were artificially reared. For each animal, 2.5 mL of whole blood was collected into PAXgene® Blood RNA tubes (BD Biosciences, USA), mixed by inversion, incubated at room temperature for 30 min, and then transported on dry ice. Total RNA extraction was performed by Personal Biotechnology Co.,Ltd. (Shanghai, China) using the Trizol reagent (Invitrogen, USA). RNA integrity was initially assessed by 1% agarose gel electrophoresis, and RNA purity and concentration were measured using a NanoDrop ND-2000 spectrophotometer (Thermo Scientific, USA).

### Library preparation, sequencing, and De Novo assembly

For RNA samples of sufficient quality, TruSeq RNA Sample Preparation Kit (Illumina, USA) was used to generate sequencing libraries. Briefly, polyadenylated mRNA was enriched from total RNA using oligo(dT) magnetic beads, fragmented into 200–300 bp fragments via ion hydrolysis, and reverse-transcribed using random oligonucleotides to construct cDNA libraries. Libraries passing quality control were subjected to paired-end sequencing (2 × 150 bp) on the Illumina NovaSeq 6000 platform (Illumina, USA) by Personal Biotechnology Co.,Ltd. (Shanghai, China).

Raw FASTQ reads were processed to remove adapter sequences at the 3' ends using the fastp tool (v0.22.0), and reads shorter than 50 bp or with average quality scores below Q20 were discarded. High-quality reads were assembled de novo using the Trinity software (v2.15.1) based on the De Bruijn Graph algorithm. Transcripts were clustered, and the longest transcript in each cluster was selected as the representative unigene.

### Functional annotation and expression analysis

Unigenes were functionally annotated using multiple databases, including NR, Gene Ontology (GO), Kyoto Encyclopedia of Genes and Genomes (KEGG), eggNOG, SwissProt, and Pfam. GO annotation was performed using BLAST2GO with default parameters, and KEGG Orthology annotations were conducted using the KOBAS annotation platform (http://kobas.cbi.pku.edu.cn/), with species categories selected according to taxonomic group. NR, Swiss-Prot, eggNOG, and Pfam annotations were performed using DIAMOND BLAST. Subsequent expression analysis was performed on the entire expression matrix, including transcripts without annotation.

Gene expression levels were quantified using RSEM (v2.15). Briefly, filtered reads from each sample were aligned to the reference transcriptome, and gene-level expression was calculated to generate the fragments per kilobase of exon per million mapped reads (FPKM) matrices. Sample correlation analyses were conducted using the DESeq R package (v1.38.3), including FPKM density distribution comparison, Pearson correlation test, and principal component analysis (PCA).

### Differential expression and pathway enrichment analysis

Differential expression analysis between naturally and artificially reared groups was performed using the DESeq R package. Differentially expressed genes (DEGs) were identified with thresholds of |log₂FoldChange|> 1 and adjusted *P*-value (Benjamini–Hochberg corrected) < 0.05. Volcano plots of DEGs were generated using the ggplot2 R package. Bidirectional hierarchical clustering on the samples and DEGs was performed using the pheatmap R package (v1.0.12), comparing the Euclidean distance and employing complete linkage as the clustering method.

GO enrichment analysis was conducted using the topGO R package (v2.50.0), with *P*-values calculated via the hypergeometric test (significance threshold: *P* < 0.05), to identify significantly enriched GO terms and infer biological functions. KEGG pathway enrichment was performed using the clusterProfiler R package (v4.6.0). Significance was first assessed by nominal *P*-values, and subsequently corrected for multiple testing using the Benjamini–Hochberg false discovery rate (FDR) procedure. Unless otherwise specified, pathways with FDR < 0.05 were considered significant, and those with *P* < 0.05 but FDR > 0.05 were reported as nominally significant and interpreted with caution. Enrichment magnitude was further assessed using the Rich Factor, defined as the ratio of DEGs enriched in a specific GO/KEGG term to the total number of genes included in the pathway.

Protein–protein interaction (PPI) analysis of DEGs was performed using the STRING database (v12.0; https://string-db.org/). Homologous protein sequences from closely related taxa were compared to infer potential interactions, and interaction pairs were retrieved in which both nodes were DEGs and the combined score exceeded 0.95. The resulting network was visualized according to expression direction (up- or down-regulated) to identify functionally associated gene groups.

## Results

### RNA extraction and sequencing

Total RNA extracted from 10 hog deer blood samples showed OD260/280 ratios of 1.800–1.894 and OD260/230 ratios of 1.802–1.892. RNA yields ranged from 0.221 to 0.318 μg (NanoDrop: 11.02–15.88), meeting sequencing requirements.

After quality control, transcriptome sequencing of 10 samples generated 84.326 Gb of data, with > 97.81% high-quality reads (Table 1). The proportion of N bases was ≤ 0.179%, and Q30 scores exceeded 96.88%, indicating good sequencing quality.

**Table 1:** Individual information and sequencing quality of 10 hog deer whole blood samples

Sample	Sex	Age (month)	Clean Data %	N (%)	GC (%)	Q20 (%)	Q30 (%)
shzapn1	Male	~ 48	97.81	0.178622	45.58	99.02	96.96
shzapn2	Male	23	98.07	0.178299	45.90	99.02	97.02
shzapn3	Male	23	98.12	0.178273	45.24	99.04	97.05
shzapn4	Female	40	98.12	0.178349	45.74	99.01	97
shzapn5	Female	36	98.23	0.178344	45.46	99.05	97.05
shzapn6	Female	28	98.22	0.178643	45.93	99.05	97.05
shzapa1	Male	13	98.09	0.178437	48.01	98.98	96.88
shzapa2	Male	10	98.26	0.178219	45.47	99.11	97.25
shzapa3	Female	14	98.23	0.178192	45.59	99.07	97.11
shzapa4	Female	4	98.53	0.172640	45.47	99.06	97.02

*bp* Base Pair, *NA* Not Available

shzapa, hog deer (Axis porcinus) artificially reared at the Shanghai Zoo; shzapn, hog deer naturally reared at the Shanghai Zoo. Age of naturally bred male shzapn1 was estimated from antler length and morphology

**Fig. 1:**
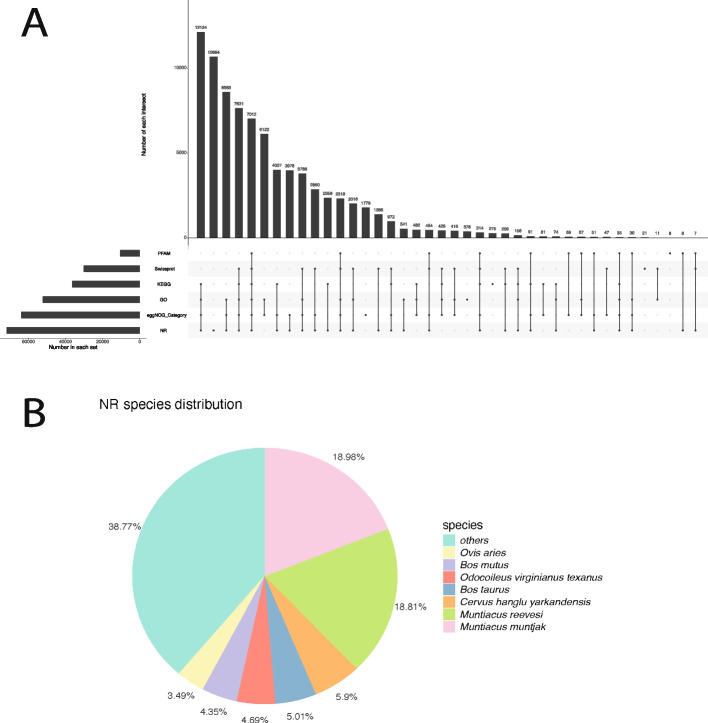
Summary of unigene annotation results. **A** UpSet plot showing the numbers of unigenes uniquely or commonly annotated by different databases. **B** Proportions of homologous sequences from different reference species contributing to NR database annotation. NR, NCBI non-redundant database

Following de novo assembly using Trinity, a total of 302,500 transcripts were obtained from the 10 samples, from which 178,336 unigenes were extracted, with an average length of 1,047 bp. Functional annotation was primarily performed using DIAMOND BLAST searches (E-value ≤ 1e − 5, more-sensitive mode) optimized for nucleotide alignment in a non-reference species. The highest number of unigenes were annotated in the NR database (71,727 unigenes, 40.22%), followed by eggNOG (63,737), GO (52,212), KEGG (36,324), and SwissProt (30,113), with the fewest matches in Pfam (10,469). A total of 7,012 unigenes (3.93%) were annotated across all six databases, and 81,879 unigenes (45.9%) were annotated in at least one database (Fig. 1A).

Due to the lack of a reference genome for hog deer, functional annotation mainly relied on homologous sequences from closely related species. The most frequently referenced genomes from the NR database were those of the southern red muntjac (*Muntiacus muntjak*) and Reeves's muntjac (*Muntiacus reevesi*)—both belonging to the family Cervidae—which accounted for 18.98% and 18.81% of annotated unigenes, respectively. Other referenced species included the Yarkend deer (*Cervus hanglu yarkandensis*, family Cervidae), domestic cattle (*Bos taurus*, family Bovidae), Texas white-tailed deer (*Odocoileus virginianus texanus*, family Cervidae), wild yak (*Bos mutus*, family Bovidae), and domestic sheep (*Ovis aries*, family Bovidae) (Fig. 1B).

### Sample correlation analysis

**Fig. 2:**
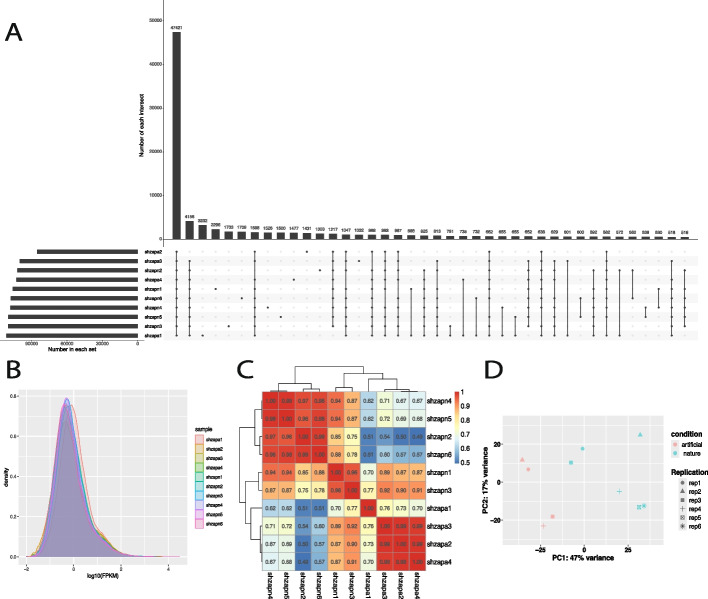
Comparison of transcript identity and expression patterns among samples. **A** UpSet plot of unique and shared mRNAs detected in different samples. **B** Expression density distribution. **C** Pairwise Pearson’s correlation of gene expression profiles. **D** Principal component analysis showing separation between artificially and naturally reared groups. FPKM, fragments per kilobase of exon per million mapped reads; PC, principal component; rep, replicate; shzapa, artificially reared hog deer (*Axis porcinus*) at the Shanghai Zoo; shzapn, naturally reared hog deer at the Shanghai Zoo

As shown in Fig. 2A, a high degree of mRNA overlap (47,421 transcripts) was observed across all samples, providing a reliable foundation for subsequent differential expression analysis. The gene expression density distribution (Fig. 2B) indicated that expression levels in all samples were mainly concentrated within the range of log₁₀(FPKM) − 2.0 to 2.0, suggesting good sequencing quality suitable for downstream analyses.

Pearson correlation analysis of expression patterns revealed r^2^ values ranging from 0.49 to 0.99 (Fig. 2C), with higher pairwise r^2^ values observed from samples within each group than between the two groups. PCA further supported the correlation patterns (Fig. 2D), with the first principal component explaining 47% of the variance, effectively distinguishing naturally and artificially reared groups. However, two samples from the natural group—shzapn1 (male, ~ 48 months) and shzapn3 (male, 23 months)—exhibited relatively distinct expression patterns. Interestingly, the second principal component appeared to distinguish samples by sex.

### Differential expression and pathway enrichment analysis

**Fig. 3:**
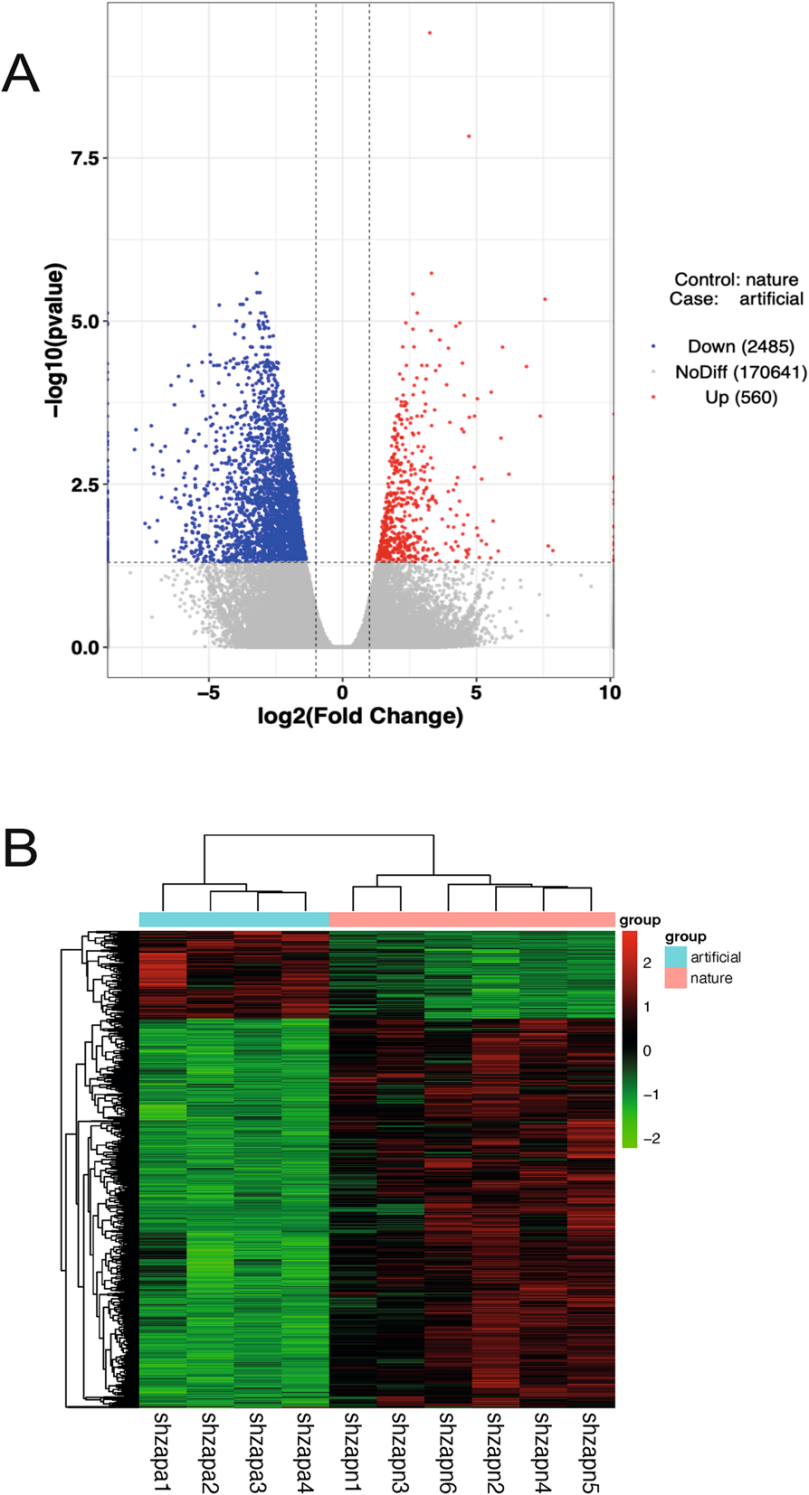
Differentially expressed genes between artificially and naturally reared hog deer. **A** Volcano plot of differentially expressed genes. **B** Heatmap of differentially expressed genes clustered by naturally and artificially reared groups. Down, downregulated; Up, upregulated; NoDiff, no significant difference; shzapa, artificially reared hog deer (*Axis porcinus*) at the Shanghai Zoo; shzapn, naturally reared hog deer at the Shanghai Zoo

As visualized in the volcano plot (Fig. 3A), a total of 3,045 DEGs were identified between artificially and naturally reared hog deer, including 560 upregulated and 2,485 downregulated genes in the artificially reared group. Hierarchical clustering of significantly differentially expressed mRNAs (Fig. 3B) revealed distinct gene expression patterns between the two rearing groups, consistent with the correlation and PCA results described above.

**Fig. 4:**
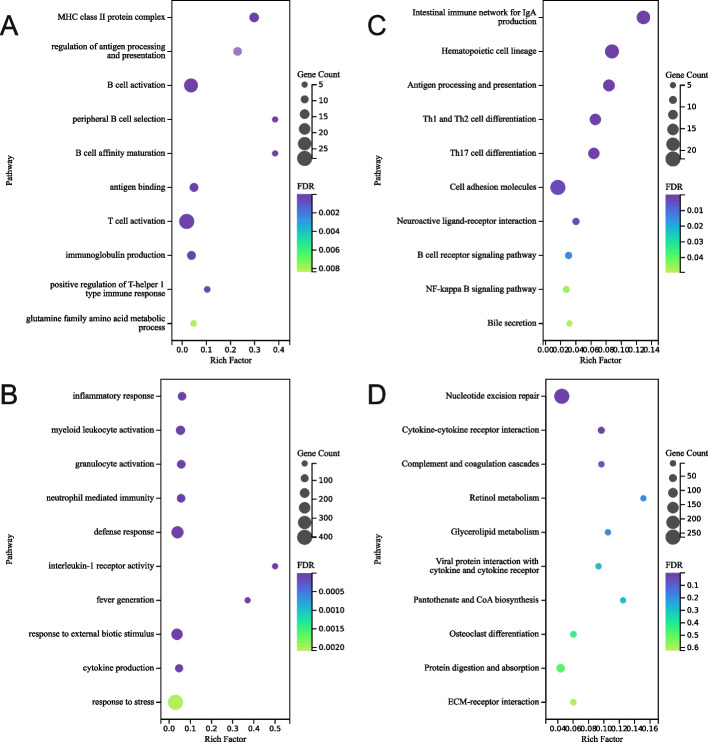
Top ten pathways with significant enrichment of up-regulated (**A** GO analysis; **C** KEGG analysis) and down-regulated genes (**B** GO; **D** KEGG) in artificially reared hog deer. The color of the dots indicates the significance of the enrichment, and the size of the dots indicates the number of genes annotated to the pathway. Rich factor is defined as the ratio of genes enriched in a specific GO/KEGG term to the total number of genes included in the pathway. CoA, co-enzyme A; ECM, extra-cellular matrix; FDR, false discovery rate; IgA, immunoglobulin A; MHC, major histocompatibility complex; Th cell, helper T cell

To investigate the signaling pathways and physiological processes associated with the DEGs, GO and KEGG enrichment analyses were performed for DEGs in artificially reared hog deer. GO analysis showed that upregulated genes in the artificial group were significantly enriched in categories related to adaptive immunity, including pathways in T/B cell development and activation, class II major histocompatibility complex (MHC) production, antigen presentation and binding, and immunoglobulin production (FDR < 0.05; Fig. 4A). Metabolic process such as glutamine family amino acid metabolism was also significantly upregulated. Downregulated genes were significantly enriched in pathways related to innate immunity and inflammatory responses, including myeloid leukocyte activation, granulocyte activation, neutrophil-mediated immunity, cytokine production, and fever generation. Notably, stress response pathways such as responses to external biotic stimuli and stress were also down-regulated (FDR < 0.05; Fig. 4B).

KEGG pathway analysis further supported the findings: upregulated genes were enriched in pathways such as intestinal immunoglobulin A production, type 1/2 helper T cell differentiation, and antigen processing and presentation. Bile secretion was also upregulated (FDR < 0.05; Fig. 4C). In contrast, downregulated genes were predominantly enriched in the cytokine–cytokine receptor interaction pathway and in the nucleotide excision repair (NER) pathway (FDR < 0.05; Fig. 4D). Additional pathways, such as the complement and coagulation cascade pathway showed nominal enrichment (P < 0.05) but did not remain significant after FDR correction.

### Protein–protein interaction analysis

**Fig. 5:**
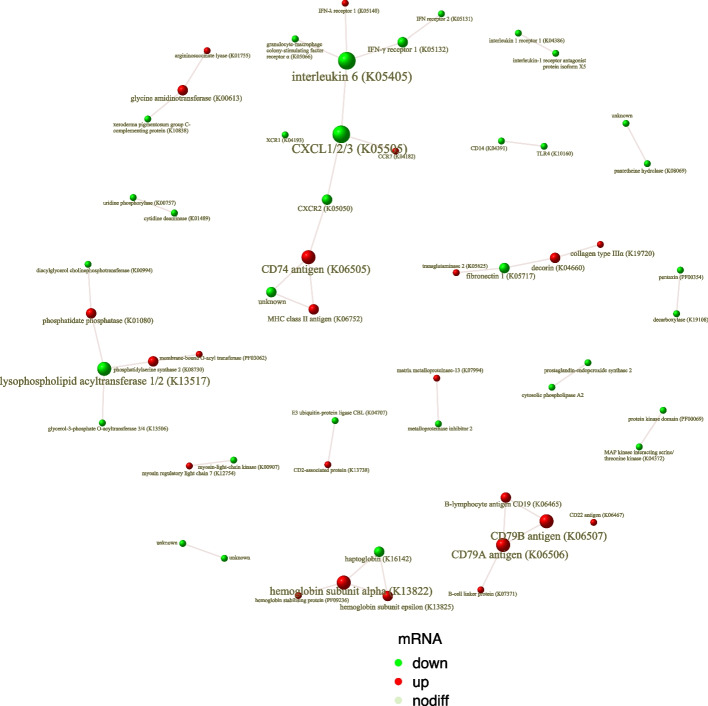
Protein–protein interaction (PPI) network of differentially expressed genes (DEGs). The network was generated using the STRING database (score > 0.95). Nodes represent DEGs, and edges indicate predicted functional associations. Node color denotes expression direction (red, up-regulated; green, down-regulated), and node size reflects expression magnitude (|log₂FoldChange|). DEGs were manually annotated according to KEGG ID wherever possible; for genes without KEGG annotation, other database identifiers were used, when available, to infer functional roles. IFN, interferon; MHC, major histocompatibility complex; TLR, toll-like receptor

To explore potential functional interactions among DEGs, a STRING network analysis was also performed (Fig. 5). In artificially reared hog deer, a major cluster showed downregulation of interleukin-6 (IL-6), CXCL1/2/3, and CXCR2, alongside upregulation of CD74 and MHC class II antigens, suggesting a shift in immune signaling. Another cluster, centered on upregulated CD79A/B, CD19, and CD22, was associated with enhanced B cell signaling and regulation. Toll-like receptor (TLR4) and CD14 were also coordinately downregulated.

Other interacting modules included downregulation of xeroderma pigmentosum complementation group C (XPC) with concurrent upregulation of glycine amidinotransferase (GATM) and argininosuccinate lyase (ASL), downregulation of lysophospholipid acyltransferase (LPAT) alongside upregulation of phosphatidylserine synthase 2 and membrane-bound O-acyl transferase (MBOAT), and upregulation of hemoglobin with downregulation of haptoglobin, suggesting potential alterations in cellular metabolism and stress response.

In addition, several genes that encode transcription factors were differentially expressed, including *SOX4*, *SOX6*, *SPIB*, and *EBF1* (upregulated in the artificial group), as well as *TFEC*, *NF-E2*, *TCF7L2*, *ELF4*, *SP3*, *GTF2IRD2B*, *E2F3*, *FOSL2*, and *ETV6* (downregulated), suggesting potential regulatory involvement in the observed differential expression profiles.

## Discussion

Genomic research on hog deer (*Axis porcinus*) is still limited, with no annotated reference genome currently available [2]. Previous transcriptome studies by Liu et al. [5] and Sui et al. [18] annotated hog deer unigenes using de novo assembly combined with multiple databases, with the highest annotation rates observed in the NR database (37.93% and 37.41%, respectively). In our study, the NR annotation rate further increased to 40.22%, likely due to the inclusion of more closely related species in recent database updates. For example, while only 12.9% of unigenes in the study of Sui et al. were annotated to Cervidae species [18], over 48% of annotated sequences in our dataset aligned with deer genomes, reflecting improved reference accuracy.

Expression pattern analyses showed distinct differences between artificially and naturally reared individuals. Pearson correlation and PCA suggested a clear separation between the two groups, which was also supported by clustering of the DEGs. Two samples from the natural group (shzapn1 and shzapn3) showed relatively distinct patterns, possibly due to individual variation rather than age or sex.

As RNA from whole blood samples mainly originates from leukocytes, blood transcriptomes are particularly useful for studying immune function [13, 14, 18–22], and are increasingly applied in stress and metabolism research [15, 20, 23]. In this study, functional enrichment analyses revealed significant intergroup differences in immunity, stress responses, and metabolism between naturally and artificially reared hog deer.

Colostrum, rich in maternal immunoglobulins, especially immunoglobulin G, provides essential passive immunity to neonates [13]. Artificially reared fawns at the Shanghai Zoo are typically switched to milk replacer within three days of birth and given prophylactic antibiotics. Inadequate colostrum intake may compromise their immune development and thus lower survival rates [18, 24]. However, our findings suggest that immune function in artificially reared individuals was not impaired but instead altered. Specifically, adaptive immunity-related modules—such as antigen presentation (class II MHC and CD74) [25] and B cell maturation (CD79/CD19)—were significantly upregulated, possibly reflecting a compensatory response to environmental antigens in the absence of maternal passive immunity [26]. Moreover, the transcription factor genes *EBF1* and *SPIB*, although not directly included in PPI modules, are canonical regulators of B-cell lineage commitment and were also upregulated in the artificial group [27, 28]. Similarly, the CD8-associated transcription factor gene *SOX4* was upregulated [29]. Conversely, innate immune responses, including phagocyte activation and cytokine-mediated pathways, were downregulated, suggesting a dampened inflammatory response. Within the downregulated modules of the PPI network, IL-1β and IL-6, key cytokines produced at sites of inflammation, promote the induction CXC family chemokines, which are potent drivers of neutrophil chemotaxis [30–33]. The observed suppression of the IL-6 axis could relieve its inhibitory effect on MHC class II expression, consistent with the upregulation of MHC class II molecules in the network analysis [34]. TLR4, a pattern recognition receptor that relies on CD14 for internalization and downstream signaling leading to IL-1β and IL-6 secretion [32, 33], was also coordinately downregulated [35, 36]. Meanwhile, the upregulation of *SOX4*, which was also reported as a transcriptional repressor of TLR signaling and pro-inflammatory cytokine production [37], may further contribute to the observed downregulation of innate immune genes. Together, these changes indicate a globally reduced inflammatory response in the artificial group. While this may reduce inflammation-induced damage, it could also imply weaker immune surveillance and slower early pathogen detection. These findings highlight the potential importance and efficacy of vaccination and other active immunization strategies in artificially reared hog deer [38, 39]. Previous work by Sui et al. using poly(I:C) to mimic viral infection showed that innate immune responses dominated the hog deer’s cellular immune defense [18], highlighting the need for future studies to explore whether artificial rearing alters such immune response profile.

Age differences between natural (mean age: 33.0 months, range: 23–48 months) and artificial (mean age: 10.3 months, range: 4–14 months) groups were also acknowledged. *FOSL2*, which was significantly downregulated in the artificial group, encodes a component of the AP-1 transcription factor complex whose expression has been reported to increase with age [40]. This suggests that age-related immune remodeling, including AP-1-driven chromatin accessibility changes and differential maturation of B- and T-cell transcriptional programs [40, 41] may partially underlie the observed expression patterns in our dataset. Indeed, potential reduction of CD8 + T cells in the older naturally reared group, supported by downregulated *SOX4*, can be attributed to age, as adaptive immunity typically declines (“immunosenescence”) and innate immunity tends to increase (“inflammaging”) with aging [41–43]. However, some studies report declining or stable innate immunity with age, depending on the specific age range examined [44, 45]. While comparisons were generally made between groups of larger age difference, in this study, differences were mainly between young and adult individuals, where sexual maturation (~ 12 months in hog deer [46]) could be a relevant factor, yet studies addressing this transition stage remain scarce. Reztak et al. reported an innate-to-adaptive immune shift in female humans during puberty [47]. In cattle, T cell abundance is highest at the neonatal stage but declines below adult levels by 3–5 months [48, 49], whereas B cells increase through 0–7 months while neutrophils decline over the same period; however, observations beyond this window are limited and transcriptomic evidence is lacking [48]. Thus, the precise contribution of age to the immunological differences observed here warrants further investigation, particularly in relation to puberty-associated immune remodelling in hog deer.

Stress responses are critical factors impacting captive animal health, with chronic stress linked to immunosuppression and reduced reproductive performance, and acute stress potentially causing sudden death [50, 51]. In this study, GO analysis showed significant downregulation of genes involved in stress responses in the artificial group, suggesting better adaptation to the captive environment, where stress is typically heightened [52, 53]. This could be attributed to closer human contact during development, despite isolation from the herd. KEGG analysis supported this at the molecular level, with significant downregulation of the NER pathway, which responds to oxidative stress and repairs DNA damage [54]. In the PPI network, the key NER initiator XPC [55], was downregulated in the artificial group, accompanied by upregulation of GATM and ASL, enzymes involved in arginine and creatine biosynthesis [56, 57]. Elevated creatine levels may enhance cellular energy buffering capacity, potentially maintaining redox and genomic stability without relying heavily on NER pathways [58]. However, the exact molecular mechanisms require functional validation. Stressful environments can also shift cell states toward pro-inflammatory gene regulation [59]. Davis et al. proposed that the neutrophil-to-lymphocyte ratio can serve as a stress biomarker [60], and Sui et al. also observed coordination between innate immunity and stress response pathways under poly(I:C) stimulation in hog deer cell [18]. The observed lower inflammatory and stress response profiles in artificially reared individuals may therefore reflect a synergistic health advantage.

Age may also influence stress reactivity. For example, aged molluscan neurons display upregulated stress response genes compared to those of sexually mature individuals [61], while hormonally prepubertal rats exhibit heightened hormonal stress reactivity relative to adults [62]. Further studies are needed to disentangle whether the weaker stress response observed in artificially reared hog deer reflects rearing environment, age, or an interaction between both factors.

Differences in metabolic pathways may reflect developmental effects of different feeding practices. Although both groups received similar feeds after weaning, the composition of milk replacer and weaning strategies may affect microbiota development and subsequent rumen function [63, 64]. Hog deer begin molar development early and can gradually switch to solid feed by around 45 days [9]. In the artificial group, pellet and forage are introduced artificially as early as one month, which may have promoted enhanced glutamine family amino acid metabolism and bile acid biosynthesis, as suggested by pathway enrichment [65]. This pattern is consistent with enhanced GATM-ASL expression [66] and altered levels of lipid metabolic enzymes such as LPAT and MBOAT [67, 68]. Nonetheless, ruminant metabolic studies typically require rumen tissue for high-resolution insight, and the accuracy of transcriptomic data from blood samples may be limited. Integrating rumen microbiome data in future work will further clarify how artificial feeding shapes metabolic function.

Despite its findings, this study has several limitations. First, the mean age of artificially reared individuals (10.3 months) was lower than that of naturally reared ones (33.0 months), which may have influenced gene expression patterns—particularly immune-related ones—although the specific differences between young and adult hog deer remain uncertain. Future work sampling mature artificially reared individuals will help resolve age effects. Second, as this study was based on a de novo transcriptome, the annotation strategy (E-value threshold and sensitivity settings) represent a compromise between maximizing homologous matches and minimizing false positives. The overall annotation rate remained modest, and several unannotated DEGs, although not yet functionally characterized, represent valuable candidates for future research, such as targeted functional studies or gene knockout experiments. Future analyses will also benefit greatly from the development of a reference genome for this species. Additionally, white blood cell counts were not determined in this study, which limits our ability to distinguish whether transcriptomic differences reflect changes in cell composition or cell-intrinsic gene expression. It is therefore hoped that future studies can integrate leukocyte profiling and single-cell transcriptomic data to systematically assess the relative contributions of these factors. Finally, this study offers an exploratory transcriptomic comparison, and further validation using quantitative PCR or proteomics is needed to confirm the findings and guide artificial rearing practices.

## Conclusions

This study highlights transcriptomic differences between artificially and naturally reared hog deer. Artificial rearing was associated with enhanced adaptive immunity, reduced innate immune activation, lower stress responsiveness, and distinct metabolic features. These findings provide molecular evidence supporting the feasibility of artificial rearing, highlighting its potential in population management and reintroduction programs. Future work should combine multi-omics and functional assays to evaluate the long-term health impacts of artificial rearing and to establish a standardized assessment system for the adaptive outcomes, thereby optimizing artificial rearing protocols.

## Data Availability

All data analyzed during this study are included in the article. The raw sequence data reported in this paper have been deposited in the Genome Sequence Archive (Genomics, Proteomics & Bioinformatics 2021) in National Genomics Data Center (Nucleic Acids Res 2022), China National Center for Bioinformation/Beijing Institute of Genomics, Chinese Academy of Sciences (GSA: CRA028057).

## Abbreviations

ASL: Argininosuccinate lyase
CITES: Convention on International Trade in Endangered Species of Wild Fauna and Flora
CoA: Coenzyme A
DEG: Differentially expressed gene
ECM: Extra-cellular matrix
FDR: False discovery rate
FPKM: Fragments per kilobase of exon per million mapped reads
GATM: Glycine amidinotransferase
GO: Gene Ontology
IFN: Interferon
IgA: Immunoglobulin A
IL: Interleukin
IUCN: International Union for Conservation of Nature
KEGG: Kyoto Encyclopedia of Genes and Genomes
LPAT: Lysophospholipid acyltransferase
MBOAT: Membrane-bound O-acyl transferase
MHC: Major histocompatibility complex
NCBI: National Center for Biotechnology Information
NER: Nucleotide excision repair
NR: NCBI non-redundant database
PCA: Principal component analysis
PPI: Protein-protein interaction
shzapa: *Axis porcinus* Artificially reared at the Shanghai Zoo
shzapn: *Axis porcinus* Naturally reared at the Shanghai Zoo
TLR: Toll-like receptor
XPC: Xeroderma pigmentosum complementation group C

## References

[1] Gupta SK, Kumar A, van Berkel T, Emsens WJ, Singh B, Puls S, et al. Genetic analysis reveals a distinct lineage of hog deer (*Axis porcinus*) in Kratie Province. Cambodia J Hered. 2022;113(4):444–52. 35373825 10.1093/jhered/esac017

[2] Wang W, Yan H-J, Chen S-Y, Li Z-Z, Yi J, Niu L-L, et al. The sequence and de novo assembly of hog deer genome. Sci Data. 2019;6(1):180305. 30620341 10.1038/sdata.2018.305 PMC6326164

[3] Angom S, Tuboi C, Ghazi MGU, Badola R, Hussain SA. Demographic and genetic structure of a severely fragmented population of the endangered hog deer (*Axis porcinus*) in the Indo-Burma biodiversity hotspot. PLoS ONE. 2020;15(2):e0210382. 32027650 10.1371/journal.pone.0210382 PMC7004368

[4] Timmins R, Duckworth JW, Samba Kumar N, Anwarul Islam M, Sagar Baral H, Long B, et al. Axis porcinus. The IUCN Red List of Threatened Species. 2015;2015:e.T41784A22157664.

[5] Liu Y, Liu F, Yu J, Niu L, Liu X, Li J. De novo assembly transcriptome of blood in *Axis porcinus* reveals gender differential expression genes. Sichuan J Zool. 2022;41(6):619–27.

[6] Wang W, Yan H, Yu J, Yi J, Qu Y, Fu M, et al. Discovery of genome-wide SNPs by RAD-seq and the genetic diversity of captive hog deer (*Axis porcinus*). PLoS ONE. 2017;12(3):e0174299. 28323863 10.1371/journal.pone.0174299 PMC5360274

[7] Geng G, You Y, Liu Q. SSR Molecular Marker Development and Genetic Diversity Analysis of Axis porcinus. Chin J Widlife. 2022;43(3):816–20.

[8] Yan H, Wang W, Yi J, Niu L, Qu Y, Chen A, et al. Seasonal Dynamics of Several Hormones in Axis porcinus. Sichuan J Zool. 2018;37:62–6.

[9] Yu J, Wu K, Li H, Liu X, Mao J, Wang Q, et al. Preliminary Study on the Growth Rule of Young Captive Hog Deer. Sichuan J Zool. 2009;28(3):428–30.

[10] Hekman JP, Johnson JL, Kukekova AV. Transcriptome analysis in domesticated species: challenges and strategies. Bioinform Biol Insights. 2015;9(Suppl 4):21–31. 26917953 10.4137/BBI.S29334 PMC4756862

[11] Hrdlickova R, Toloue M, Tian B. RNA-Seq methods for transcriptome analysis. Wiley Interdiscip Rev RNA. 2017;8(1). 10.1002/wrna.1364 PMC5717752 27198714

[12] Jiang Z, Zhou X, Li R, Michal JJ, Zhang S, Dodson MV, et al. Whole transcriptome analysis with sequencing: methods, challenges and potential solutions. Cell Mol Life Sci. 2015;72(18):3425–39. 26018601 10.1007/s00018-015-1934-y PMC6233721

[13] Surlis C, Earley B, McGee M, Keogh K, Cormican P, Blackshields G, et al. Blood immune transcriptome analysis of artificially fed dairy calves and naturally suckled beef calves from birth to 7 days of age. Sci Rep. 2018;8(1):15461. 30337646 10.1038/s41598-018-33627-0 PMC6194081

[14] Yang M, Huang Y, Wu H, Li C, Ling S, Sun J, et al. Blood transcriptome analysis revealed the immune changes and immunological adaptation of wildness training giant pandas. Mol Genet Genomics. 2022;297(1):227–39. 34985592 10.1007/s00438-021-01841-7

[15] Watson H, Videvall E, Andersson MN, Isaksson C. Transcriptome analysis of a wild bird reveals physiological responses to the urban environment. Sci Rep. 2017;7:44180. 28290496 10.1038/srep44180 PMC5349542

[16] Wen J, Zhao B, Cao Y, Qu Y, Chang L, Mao J, et al. Physiology-related variations in the blood hormone and metabolome of endangered hog deer (*Axis porcinus*). Metabolites. 2025;15(2):126. 39997752 10.3390/metabo15020126 PMC11857704

[17] Grabherr MG, Haas BJ, Yassour M, Levin JZ, Thompson DA, Amit I, et al. Full-length transcriptome assembly from RNA-Seq data without a reference genome. Nat Biotechnol. 2011;29(7):644–52. 21572440 10.1038/nbt.1883 PMC3571712

[18] Sui W, Gao Y, Yang Q, Leng S, Yan H, Qu Y, et al. Transcriptome Analysis of Hog Deer (Axis Porcinus) Peripheral Blood Lymphocyte after PolyI:C Challenge. Journal of Sichuan Agricultural University. 2021;39:114–28.

[19] Du L, Liu Q, Shen F, Fan Z, Hou R, Yue B, et al. Transcriptome analysis reveals immune-related gene expression changes with age in giant panda (*Ailuropoda melanoleuca*) blood. Aging (Albany NY). 2019;11(1):249–62. 30641486 10.18632/aging.101747 PMC6339791

[20] Huang X, Ouyang Q, Ran M, Zeng B, Deng L, Hu S, et al. The immune and metabolic changes with age in giant panda blood by combined transcriptome and DNA methylation analysis. Aging (Albany NY). 2020;12(21):21777–97. 33188156 10.18632/aging.103990 PMC11623972

[21] Shen H, Li C, He M, Huang Y, Wang J, Wang M, et al. Immune profiles of male giant panda (*Ailuropoda melanoleuca*) during the breeding season. BMC Genomics. 2021;22(1):143. 33639852 10.1186/s12864-021-07456-x PMC7916315

[22] Sun X, Cai R, Jin X, Shafer ABA, Hu X, Yang S, et al. Blood transcriptomics of captive forest musk deer (*Moschus berezovskii*) and possible associations with the immune response to abscesses. Sci Rep. 2018;8(1):599. 29330436 10.1038/s41598-017-18534-0 PMC5766596

[23] Wang Y, Guo J, Wang L, Tian H, Sui J. Transcriptome analysis revealed potential mechanisms of differences in physiological stress responses between caged male and female magpies. BMC Genomics. 2019;20(1):447. 31159743 10.1186/s12864-019-5804-0 PMC6547487

[24] Deng J, Yu J, Niu L, Wang Q, Qu Y, Yan H, et al. Etiology of low survival rate of hog deer. Chin J Widlife. 2011;32(3):120–2+55.

[25] Beswick EJ, Reyes VE. CD74 in antigen presentation, inflammation, and cancers of the gastrointestinal tract. World J Gastroenterol. 2009;15(23):2855–61. 19533806 10.3748/wjg.15.2855 PMC2699002

[26] Semmes EC, Chen JL, Goswami R, Burt TD, Permar SR, Fouda GG. Understanding early-life adaptive immunity to guide interventions for pediatric health. Front Immunol. 2020;11:595297. 33552052 10.3389/fimmu.2020.595297 PMC7858666

[27] Györy I, Boller S, Nechanitzky R, Mandel E, Pott S, Liu E, et al. Transcription factor Ebf1 regulates differentiation stage-specific signaling, proliferation, and survival of B cells. Genes Dev. 2012;26(7):668–82. 22431510 10.1101/gad.187328.112 PMC3323878

[28] Horiuchi S, Koike T, Takebuchi H, Hoshino K, Sasaki I, Fukuda-Ohta Y, et al. SpiB regulates the expression of B-cell-related genes and increases the longevity of memory B cells. Frontiers in Immunology. 2023;Volume 14 - 2023. 10.3389/fimmu.2023.1250719 PMC10641807 37965309

[29] Hu G, Chen J. A genome-wide regulatory network identifies key transcription factors for memory CD8⁺ T-cell development. Nat Commun. 2013;4:2830. 24335726 10.1038/ncomms3830 PMC3999894

[30] Ahuja N, Andres-Hernando A, Altmann C, Bhargava R, Bacalja J, Webb RG, et al. Circulating IL-6 mediates lung injury via CXCL1 production after acute kidney injury in mice. Am J Physiol Renal Physiol. 2012;303(6):F864–72. 22791336 10.1152/ajprenal.00025.2012 PMC3468527

[31] Gabay C. Interleukin-6 and chronic inflammation. Arthritis Res Ther. 2006;8 Suppl 2(Suppl 2):S3. 10.1186/ar1917 PMC3226076 16899107

[32] Hou SM, Chen PC, Lin CM, Fang ML, Chi MC, Liu JF. CXCL1 contributes to IL-6 expression in osteoarthritis and rheumatoid arthritis synovial fibroblasts by CXCR2, c-Raf, MAPK, and AP-1 pathway. Arthritis Res Ther. 2020;22(1):251. 33087182 10.1186/s13075-020-02331-8 PMC7580030

[33] Lv Y, Chen C, Han M, Tian C, Song F, Feng S, et al. CXCL2: a key player in the tumor microenvironment and inflammatory diseases. Cancer Cell Int. 2025;25(1):133. 40197328 10.1186/s12935-025-03765-3 PMC11978139

[34] Kitamura H, Kamon H, Sawa S, Park SJ, Katunuma N, Ishihara K, et al. IL-6-STAT3 controls intracellular MHC class II alphabeta dimer level through cathepsin S activity in dendritic cells. Immunity. 2005;23(5):491–502. 16286017 10.1016/j.immuni.2005.09.010

[35] Chávez-Sánchez L, Chávez-Rueda K, Legorreta-Haquet MV, Zenteno E, Ledesma-Soto Y, Montoya-Díaz E, et al. The activation of CD14, TLR4, and TLR2 by mmLDL induces IL-1β, IL-6, and IL-10 secretion in human monocytes and macrophages. Lipids Health Dis. 2010;9:117. 20946675 10.1186/1476-511X-9-117 PMC2964726

[36] Ciesielska A, Matyjek M, Kwiatkowska K. TLR4 and CD14 trafficking and its influence on LPS-induced pro-inflammatory signaling. Cell Mol Life Sci. 2021;78(4):1233–61. 33057840 10.1007/s00018-020-03656-y PMC7904555

[37] Shang J, Zheng Y, Mo J, Wang W, Luo Z, Li Y, et al. Sox4 represses host innate immunity to facilitate pathogen infection by hijacking the TLR signaling networks. Virulence. 2021;12(1):704–22. 33517839 10.1080/21505594.2021.1882775 PMC7894441

[38] Palmer MV, Kanipe C, Lehman KA, Thacker TC, Putz EJ, Boggiatto PM. Vaccination of white-tailed deer with *Mycobacterium bovis* Bacillus Calmette-Guérin (BCG): effect of *Mycobacterium avium* ssp. *paratuberculosis* infection. Microorganisms. 2023. 10.3390/microorganisms11102488. 37894146 10.3390/microorganisms11102488 PMC10609214

[39] Nol P, Palmer MV, Waters WR, Aldwell FE, Buddle BM, Triantis JM, et al. Efficacy of oral and parenteral routes of *Mycobacterium bovis* bacille Calmette-Guerin vaccination against experimental bovine tuberculosis in white-tailed deer (*Odocoileus virginianus*): a feasibility study. J Wildl Dis. 2008;44(2):247–59. 18436658 10.7589/0090-3558-44.2.247

[40] Lynch CJ, Richart L, Serrano M. A pattern emerges in chromatin aging: AP-1 steals the show. Cell Metab. 2024;36(8):1639–41. 39111283 10.1016/j.cmet.2024.07.015

[41] Morandini F, Rechsteiner C, Perez K, Praz V, Lopez Garcia G, Hinte LC, et al. ATAC-clock: An aging clock based on chromatin accessibility. GeroScience. 2024;46(2):1789–806. 37924441 10.1007/s11357-023-00986-0 PMC10828344

[42] Santoro A, Bientinesi E, Monti D. Immunosenescence and inflammaging in the aging process: age-related diseases or longevity? Ageing Res Rev. 2021;71:101422. 34391943 10.1016/j.arr.2021.101422

[43] Buggiotti L, Cheng Z, Salavati M, Wathes CD, Fahey A, Crisà A, et al. Comparison of the transcriptome in circulating leukocytes in early lactation between primiparous and multiparous cows provides evidence for age-related changes. BMC Genomics. 2021;22(1):693. 34563126 10.1186/s12864-021-07977-5 PMC8466696

[44] Gao H, Nepovimova E, Adam V, Heger Z, Valko M, Wu Q, et al. Age-associated changes in innate and adaptive immunity: role of the gut microbiota. Front Immunol. 2024;15:1421062. 39351234 10.3389/fimmu.2024.1421062 PMC11439693

[45] Chase CC, Hurley DJ, Reber AJ. Neonatal immune development in the calf and its impact on vaccine response. Vet Clin North Am Food Anim Pract. 2008;24(1):87–104. 18299033 10.1016/j.cvfa.2007.11.001 PMC7127081

[46] Hill E, Murphy N, Linacre A, Toop S, Strugnell JM. Kinship analysis reveals low dispersal in a hog deer (*Axis porcinus*) population in Wilsons Promontory National Park. Australia Wildlife Research. 2023;50(9):746–56.

[47] Resztak JA, Choe J, Nirmalan S, Wei J, Bruinsma J, Houpt R, et al. Analysis of transcriptional changes in the immune system associated with pubertal development in a longitudinal cohort of children with asthma. Nat Commun. 2023;14(1):230. 36646693 10.1038/s41467-022-35742-z PMC9842661

[48] Kampen AH, Olsen I, Tollersrud T, Storset AK, Lund A. Lymphocyte subpopulations and neutrophil function in calves during the first 6 months of life. Vet Immunol Immunopathol. 2006;113(1–2):53–63. 16772096 10.1016/j.vetimm.2006.04.001

[49] Wilson RA, Zolnai A, Rudas P, Frenyo LV. T-cell subsets in blood and lymphoid tissues obtained from fetal calves, maturing calves, and adult bovine. Vet Immunol Immunopathol. 1996;53(1–2):49–60. 8941968 10.1016/0165-2427(95)05543-6

[50] Fischer CP, Romero LM. Chronic captivity stress in wild animals is highly species-specific. Conserv Physiol. 2019. 10.1093/conphys/coz093. 31824674 10.1093/conphys/coz093 PMC6892464

[51] Zhang F, Tan Y, Cai Z, An K, Liu Y, Su J. Two plants improve stress response of a subterranean herbivore by downregulating amphetamine addiction pathways. Front Vet Sci. 2024;10:1342630. 38283372 10.3389/fvets.2023.1342630 PMC10811048

[52] Bedoya Duque MA, Thomas WR, Dechmann DKN, Nieland J, Baldoni C, von Elverfeldt D, et al. Gene expression comparisons between captive and wild shrew brains reveal captivity effects. Biol Let. 2025;21(1):20240478. 39772919 10.1098/rsbl.2024.0478 PMC11706642

[53] Morin M, Jönsson M, Wang CK, Craik DJ, Degnan SM, Degnan BM. Captivity induces a sweeping and sustained genomic response in a starfish. Mol Ecol. 2023;32(13):3541–56. 37009965 10.1111/mec.16947

[54] Chien L-C, Wu Y-H, Ho T-N, Huang Y-Y, Hsu T. Heat stress modulates nucleotide excision repair capacity in zebrafish (*Danio rerio*) early and mid-early embryos via distinct mechanisms. Chemosphere. 2020;238:124653. 31473528 10.1016/j.chemosphere.2019.124653

[55] Menoni H, Hoeijmakers JHJ, Vermeulen W. Nucleotide excision repair–initiating proteins bind to oxidative DNA lesions in vivo. J Cell Biol. 2012;199(7):1037–46. 23253478 10.1083/jcb.201205149 PMC3529521

[56] Erez A, Nagamani SCS, Lee B. Argininosuccinate lyase deficiency—argininosuccinic aciduria and beyond. Am J Med Genet C Semin Med Genet. 2011;157(1):45–53. 10.1002/ajmg.c.30289 PMC3073162 21312326

[57] Baker SA, Gajera CR, Wawro AM, Corces MR, Montine TJ. GATM and GAMT synthesize creatine locally throughout the mammalian body and within oligodendrocytes of the brain. Brain Res. 2021;1770:147627. 34418357 10.1016/j.brainres.2021.147627

[58] Mahajan AS, Arikatla VS, Thyagarajan A, Zhelay T, Sahu RP, Kemp MG, et al. Creatine and nicotinamide prevent oxidant-induced senescence in human fibroblasts. Nutrients. 2021. 10.3390/nu13114102. 34836359 10.3390/nu13114102 PMC8622652

[59] Díaz-Bulnes P, Saiz ML, López-Larrea C, Rodríguez RM. Crosstalk Between Hypoxia and ER Stress Response: A Key Regulator of Macrophage Polarization. Frontiers in Immunology. 2020;Volume 10 - 2019. 10.3389/fimmu.2019.02951 PMC6961549 31998288

[60] Davis AK, Maney DL, Maerz JC. The use of leukocyte profiles to measure stress in vertebrates: a review for ecologists. Funct Ecol. 2008;22(5):760–72.

[61] Greer JB, Schmale MC, Fieber LA. Whole-transcriptome changes in gene expression accompany aging of sensory neurons in *Aplysia californica*. BMC Genomics. 2018;19(1):529. 29996779 10.1186/s12864-018-4909-1 PMC6042401

[62] Foilb AR, Lui P, Romeo RD. The transformation of hormonal stress responses throughout puberty and adolescence. J Endocrinol. 2011;210(3):391–8. 21746793 10.1530/JOE-11-0206

[63] Wang M, Yi M, Wang L, Sun S, Ling Y, Zhang Z, et al. Multi-omics analysis reveals the regulatory mechanism of probiotics on the growth performance of fattening sheep. Animals. 2024. 10.3390/ani14091285. 38731289 10.3390/ani14091285 PMC11083020

[64] Liu T, Li F, Wang W, Wang X, Ma Z, Li C, et al. Early feeding strategies in lambs affect rumen development and growth performance, with advantages persisting for two weeks after the transition to fattening diets. Frontiers in Veterinary Science. 2022;Volume 9 - 2022. 10.3389/fvets.2022.925649 PMC9366302 35968009

[65] Dicks L, Schuh-von Graevenitz K, Prehn C, Sadri H, Murani E, Hosseini Ghaffari M, et al. Bile acid profiles and mRNA abundance of bile acid-related genes in adipose tissue of dairy cows with high versus normal body condition. J Dairy Sci. 2024;107(8):6288–307. 38490538 10.3168/jds.2024-24346

[66] Wu G, Bazer FW, Davis TA, Kim SW, Li P, Marc Rhoads J, et al. Arginine metabolism and nutrition in growth, health and disease. Amino Acids. 2009;37(1):153–68. 19030957 10.1007/s00726-008-0210-y PMC2677116

[67] Matsuda S, Inoue T, Lee HC, Kono N, Tanaka F, Gengyo-Ando K, et al. Member of the membrane-bound O-acyltransferase (MBOAT) family encodes a lysophospholipid acyltransferase with broad substrate specificity. Genes Cells. 2008;13(8):879–88. 18782225 10.1111/j.1365-2443.2008.01212.x

[68] Pierce MR, Hougland JL. A rising tide lifts all MBOATs: recent progress in structural and functional understanding of membrane bound O-acyltransferases. Front Physiol. 2023;14:1167873. 37250116 10.3389/fphys.2023.1167873 PMC10213974

